# Cytokine and Lymphocyte Profiles in Dogs with Atopic Dermatitis after Allergen-Specific Immunotherapy

**DOI:** 10.3390/vaccines10071037

**Published:** 2022-06-28

**Authors:** Alicja Majewska, Kourou Dembele, Katarzyna Dziendzikowska, Adam Prostek, Małgorzata Gajewska

**Affiliations:** 1 Department of Physiological Sciences, Institute of Veterinary Medicine, Warsaw University of Life Sciences (SGGW), Nowoursynowska 159, 02-776 Warsaw, Poland; adam_prostek@sggw.edu.pl (A.P.); malgorzata_gajewska@sggw.edu.pl (M.G.); 2 Department of Small Animal Diseases and Clinic, Institute of Veterinary Medicine, Warsaw University of Life Sciences (SGGW), Nowoursynowska 159, 02-776 Warsaw, Poland; kourou_dembele@sggw.edu.pl; 3 Department of Dietetics, Institute of Human Nutrition Sciences, Warsaw University of Life Sciences (SGGW), Nowoursynowska 159c, 02-776 Warsaw, Poland; katarzyna_dziendzikowska@sggw.edu.pl

**Keywords:** canine atopic dermatitis, allergen-specific immunotherapy, lymphocytes, T cells, Treg cells, cytokines, PBMC

## Abstract

Canine atopic dermatitis (cAD) is a chronic and recurrent inflammatory and pruritic skin disease in dogs. Currently, allergen-specific immunotherapy (ASIT) is the only identified disease-modifying intervention for allergic diseases. It decreases the symptoms triggered by allergens and prevents recurrence of the disease in the long-term. The aim of our research was to determine how immunotherapy changes the proportion of lymphocyte subsets in dog peripheral blood and the levels of cytokines secreted by these cells during therapy. ASIT was applied for 6 months. Blood samples for further analyses were collected from patients in the third and sixth month of immunotherapy. Six out of seven dogs receiving ASIT showed a positive effect. A reduction in cytokine levels (IL-13, TNF-α) in peripheral blood of cAD patients and changes in the number of specific T cell subpopulations—reduction of Tc cells (CD8^+^) and increase of activated T cells (CD3^+^CD25^+^)—confirmed the beneficial effect of the applied ASIT. In addition, a significantly higher percentage of Treg cells (CD4^+^CD25^+^FOXP3^+^) was noted in cAD patients before treatment compared to healthy dogs. After 3 months of therapy, the percentage of Tregs significantly decreased, and after 6 months, it increased significantly again.

## 1. Introduction

Canine atopic dermatitis (cAD) is a chronic and recurrent inflammatory and pruritic skin disease that affects 10% of the canine population. It has been defined by the International Task Force on Canine Atopic Dermatitis (ITFCAD) as a “genetically predisposed inflammatory and pruritic allergic skin disease with characteristic clinical features associated with IgE antibodies most commonly directed against environmental allergens” [1].

The initial clinical feature of cAD is pruritus. At onset, pruritus may be without skin-visible lesions or with primary skin lesions, such as erythema and occasionally papules [2,3,4]. However, Olivry and Banovic [5] stated that in dogs with AD even normal-looking skin is microscopically inflamed. The location of pruritus and skin lesions is rather characteristic. The most-often affected areas are the ear pinnae, axillae, inguinal area, abdomen, front and hind feet, lips and perineal area. Depending on the sensitivity to the allergen, symptoms may be seasonal (e.g., pollen) or not seasonal (e.g., mites). Approximately 80% of dogs with seasonal signs are symptomatic in spring or summer [4,6]. 

AD develops as a result of defective innate and adaptive immune responses and alteration of the physico–chemical properties of the epidermis. The inflammatory reaction is caused by an imbalance between T helper cells: Th2 and Th1. The initial acute T-helper 2 (Th2) response is characterized by predominant secretion of IL-4, IL-5 and IL-13 interleukins, resulting in recruitment of eosinophils into the inflammatory site, and activation of B lymphocytes, which are stimulated to produce IgE. Binding of allergen-specific IgE to mast cells causes degranulation of these cells. The secreted inflammatory mediators lead to inflammation. In human AD (hAD), the acute phase leads to the Th1-driven chronic phase. Activation of Th1 cells causes IFN-γ, IL-2 and IL-12 secretion [7]. In dogs, only the initial Th2 type response is typically found, and it is difficult to recognize the typical Th1 type response, but rather a mixed Th1–Th2 response is observed [8,9]. In addition to Th cells (CD4^+^), T cytotoxic (Tc CD8^+^) cells play a role in atopic dermatitis, and more of these cells have been recruited in atopic skin, which has been demonstrated in humans, mice and dogs [10,11,12,13,14]. In our previous research, we observed increased concentrations of IL-13 and TNF-α in the plasma of cAD patients [15]. Our studies also showed an increased number of CD8^+^ cells in the blood of atopic dogs [15]. In the abnormal response of the immune system to the allergen, attention is drawn to the insufficient response of suppressive T regulatory (Treg) cells. Treg cells’ suppressive functions are similar between controlling autoimmunity and allergy, and their action is mediated by multiple mechanisms, including the release of suppressive cytokines (IL-10, TGF-β1 and IL-35) [16]. In humans, fewer Treg cells are observed in patients with atopy, while in atopic dogs, a higher number of Treg cells are observed in comparison to healthy animals [15,17,18]. Nevertheless, it seems there may be too few Treg lymphocytes, or they may show a functional defect. 

The best treatment for AD is to avoid allergens. Unfortunately, this is impossible in most cases, so symptomatic treatment is necessary. In severely affected dogs, reactive therapy involves topical and/or oral administration of glucocorticoids, ciclosporin, oclacitinib and lokivetmab due to their clinical efficacy and high success rates of 70–80% [19,20]. It is worth remembering that these drugs may cause adverse effects. According to Olivry and Banovic [5], once the patient has remained clear of clinical signs for several weeks, it is time to move to the second phase of AD treatment—“proactive therapy”, which aims to prevent the development of flares. In the second phase allergen specific immunotherapy (ASIT) can be used. In approximately 50–75% of atopic animals, desensitization is effective [20,21]. This form of therapy typically involves subcutaneous administration of gradually increasing quantities of the patient’s relevant allergens until a dose is reached that is effective in inducing immunologic tolerance to the allergens. The primary objectives of allergen-specific immunotherapy are to decrease the symptoms triggered by allergens and to prevent recurrence of disease in the long-term. Currently, this is the only identified disease-modifying intervention for allergic disease [22,23,24].

The immune changes that occur during ASIT are complex and not entirely clear. Much more is known about immunotherapy in humans than in dogs. Effective immunotherapy in human results in a shift from the Th2 immune response to a better balance with more Th1 immune responses [23]. The goal of ASIT is to induce peripheral T cell tolerance to allergens, modulate the thresholds for mast cell and basophil activation and decrease IgE-mediated histamine release [25]. Peripheral tolerance is associated with generation of allergen-specific regulatory T cells. IL-4 secreting Th2 cells switch towards IL-10 secreting inducible Treg (iTreg) cells: FOXP3-expressing iTregs and CD4^+^FOXP3^−^ IL-10-producing Tr1 cells [26,27]. Treg cells act multi-directionally by secreting anti-inflammatory cytokines IL-10 and TGF-β, as well as by expression of cell-surface suppressive molecules, such as cytotoxic T lymphocyte antigen 4 (CTLA-4) and programmed death-1 (PD-1). They suppress allergen-specific T cell proliferation and cytokine secretion, directly or indirectly reducing activity and degranulation of effector cells, such as mast cells, basophils and eosinophils [28,29]. 

The mechanism of response to ASIT in dogs has not been studied as extensively as in humans. There are sparse data related to changes in cellular response in dogs undergoing immunotherapy. Therefore, the aim of our research was to determine how immunotherapy changes the proportion of lymphocyte subsets in peripheral blood and the cytokines secreted by these cells.

## 2. Materials and Methods

### 2.1. Animals

This study complied with national and institutional guidelines on the use of animals in clinical research according to the Polish legal act from 21 January 2005 (Ustawa o doświadczeniach na zwierzętach z dnia 21 stycznia 2005 r. (Dz. U. z 2005 r. Nr 33, poz. 289 z późn.zm.) concerning experiments performed on client-owned animals. All dogs were patients of the Small Animal Clinic at Warsaw University of Life Sciences. A high standard of care was adhered to throughout each examination. In the case of canine atopic dermatitis (cAD) patients, research was carried out as part of routine veterinary diagnostic procedures. Dogs included in the control group were blood donors from the “Milusia” Veterinary Blood Bank, who had been submitted to the Small Animal Clinic for routine checkup.

Seven privately owned dogs of various breeds with cAD (five females and two males) were included in this study. The breeds were: Labrador retriever (2), Golden retriever (2), American Staffordshire terrier (2) and Small Münsterlander (1). Their age ranged between 2 years and 2 months and 6.5 years. Eight healthy dogs served as a control group (three females and five males), their age ranging between 3 and 8 years. The following breeds were included in the control group: American Staffordshire terrier (2), Labrador retriever (2), bulldog (1), Great Dane (1), Staffordshire Bull terrier (1) and Weimaraner (1).

### 2.2. cAD Diagnosis and Sample Collection

Diagnosis of cAD was based on compatible history and clinical signs determined using Willemse and Prélaud diagnostic criteria, completed by Favrot criteria as follows: pruritus sine materia, indoor lifestyle and the exclusion of other causes of pruritus ongoing for at least one year.

In all dogs with chronic pruritus, other causative factors were excluded, i.e., skin parasites (Sarcoptic mange, Demodectic mange, flea allergic dermatitis). Bacterial pyoderma and Malassezia dermatitis were excluded by negative in vitro culture assays. The role of food antigens as a cause of the skin condition was assessed using elimination diets for 6–8 weeks. Clinical diagnosis of atopic dermatitis was confirmed by serological allergy testing (IDEXX allergic panel test) and intradermal skin testing (Artuvetrin test set, Netherlands). No anti-inflammatory drugs were given for at least 3 weeks prior to serological and intradermal tests.

All dogs in the investigated group had positive reactions to serological allergy testing and intradermal skin testing. Peripheral blood samples were collected just before the dogs were subjected to intradermal skin testing, thus at the stage when clinical signs of AD were visible, as well as after 3 months and 6 months of therapy. Hematological, morphological and biochemical blood tests were conducted on samples of qualified patients. Each dog with AD, as well as the animals included in the control group, showed morphological parameters of blood within reference value ranges.

Blood used for cytometric or transcriptomic analyses and ELISA tests was collected once and separated into portions that were then used in particular analyses. Blood samples were collected from client-owned dogs during routine veterinary examinations.

The intradermal skin test (Artuvetrin test set, Netherlands) used on the experiment dogs showed that the dogs were primarily sensitized to storage mites—Tyrophagus putrescentiae 86% (6 dogs), Acarus siro 86% (6 dogs), Lepidoglyphus destructor 57% (4 dogs)—and subsequently house dust mites—Dermatophagoides farine 71% (5 dogs), Dermatophagoides pteronyssimus 29% (2 dogs)—tree pollen mixture 43% (2 dogs), weed pollen mixture 29% (2 dogs) and grass pollen mixture 29% (2 dogs).

### 2.3. Allergen-Specific Immunotherapy (ASIT)

Allergen extracts were prepared based on the results of intradermal tests by the Artuvetrin Therapy company. Allergen extracts were administered subcutaneously in increasing concentrations according to the manufacturer’s recommendations. 

The first dosage for subcutaneous Artuvetrin Therapy started at 0.2 mL, after which it was gradually increased over longer intervals to a maximum of 1.0 mL. In the 3rd week, the dose was increased to 0.4 mL; in the 5th week, the dose was increased to 0.6 mL; in the 7th to 0.8 mL, and in 10th, 13th and 17th, the dose was 1 mL; then, 1 mL was administered every 4 weeks. In two patients, the time between doses was extended due to a hypersensitivity reaction that followed each dose of the allergen extract.

When the 1 mL dose was reached after 12 weeks, a fixed dose of 1.0 mL was administered monthly. In some cases, this treatment schedule was too fast for the patient. If so, it was possible to deviate from the standard dosage schedule. 

### 2.4. Flow Cytometric Analysis of Peripheral Blood Mononuclear Cells (PBMC) 

Peripheral blood was collected into EDTA anticoagulant tubes. PBMC were isolated from whole blood by density gradient centrifugation in histopaque-1077 using a protocol provided with the ACCUSPIN System–HISTOPAQUE-1077 (Sigma-Aldrich, St. Louis, MO, USA). 

#### 2.4.1. Analysis of Lymphocyte Subpopulations with Flow Cytometry 

Freshly isolated PBMC were used to determine the lymphocyte subpopulations by flow cytometry (FACS Aria II, BD Bioscience, San Jose, CA, USA). Two commercially available sets of antibodies were used. Dog T Lymphocyte cocktail (BD PharmingenTM, San Jose, NJ, USA) served to determine the number of T, Th (T helper) and Tc (T cytotoxic) cells and was comprised of: APC-conjugated anti-CD3 and PE-conjugated anti-CD4 and FITC-conjugated anti-CD8 antibodies. Dog activated T lymphocyte cocktail (BD PharmingenTM, San Jose, NJ, USA) was used to determine the number of activated T cells and B lymphocytes, and included 3 antibodies: APC-conjugated anti-CD3, FITC-conjugated clone CTL 2.58 generated by using whole cell immunizations of IL-2 dependent feline T cell lines stimulated with PHA and Con A and reacting with dog T cell activation marker, and PE-conjugated LSM 11.425 antibody generated against cells derived from canine peripheral lymph nodes and used as a prognostic tool in dog B cell lymphoma studies. 

PBMC were suspended in 100 μL of phosphate buffered saline (PBS), and 10 μL of appropriate antibodies were added according to the manufacturer’s instructions. Cells were incubated at room temperature for 30 min in the dark; next, the cells were washed in PBS and analyzed using BD FACSAria™ II flow cytometer (BD Biosciences, San Jose, NJ, USA). Data were collected from 20,000 lymphocytes. The population of lymphocytes was first gated based on morphological characteristics: forward scatter (FSC) and side scatter (SSC) (gate P1). Cells located in gate P1 were then analyzed with regard to their positive staining with appropriate antibodies. Unstained cells were used as negative control. 

#### 2.4.2. Treg Cell Analysis with Flow Cytometry

In order to determine the number of Treg lymphocytes, freshly isolated PBMC were first stained for 30 min with antibodies against two surface markers: APC-conjugated anti-CD4 monoclonal antibody (mAb, clone: YKIX302.9; eBioscience, San Diego, CA, USA) and FITC-conjugated anti-canine CD25 mAb (clone: P4A10; eBioscience, San Diego, CA, USA). Appropriate isotypic controls (rat IgG2a: APC, mouse IgG1:FITC) were used as negative controls. Then, cells were permeabilized in fixation/permeabilization buffer for 18 h at 4 °C in the dark. After incubation, cells were stained intracellularly for FOXP3 for 30 min using cross-reactive, directly conjugated anti-mouse/rat FOXP3 PE mAb (clone: FJK-16s; eBioscience, San Diego, CA, USA) or isotype control (Rat IgG2a: PE). Fixation and permabilization of cells was performed using a set of buffers (FOXP3/Transcription Factor Staining Buffer Set, eBioscience, San Diego, CA, USA) recommended by the producer (eBioscience) for FOXP3 staining. The stained cells were analyzed using flow cytometry. 

### 2.5. Measurement of Cytokine Concentration in Plasma by ELISA 

The concentration of cytokines IL-2, IL-4, IL-10, IL-13, TNF-α TGF-β 1and IFN-γ in plasma were determined by ELISA. For all cytokines except IFN-γ, dog-specific tests from USCN Life Science (CLOUD-CLONE CORP., Wuhan, China) were used, and IFN-γ concentration was determined using Canine IFN-gamma Quantikine ELISA Kit (R&D System, Minneapolis, MN, USA). The detection limit was 5.6 pg/mL for IL-13, 5.6 pg/mL for IL-4, 5.8 pg/mL for IL-2, 5.9 pg/mL for IL-10, 6.2 pg/mL for TNF-α and TGF-β 1, and 60 pg/mL for IFN-γ. ELISAs were performed according to the protocols provided by the producers.

### 2.6. Statistical Analysis

Experimental data from flow cytometry and ELISA were analyzed using statistical software Statistica v13.3 (StatSoft, USA) by two-way ANOVA (treatment vs. months, with repeated measures on months) followed by Fisher’s post-hoc test. Results are presented as mean ± SD (standard deviation of the mean). Normal distribution and equality of variances were tested for all data. If data had non-normal distribution, logarithmic transformation was applied. Statistically significant difference was accepted at a level of *p* < 0.05.

## 3. Results

### 3.1. Patients

A description of the atopic dermatitis (AD) patients and their clinical symptoms are presented in Table 1. The response of AD patients to subcutaneous allergen-specific immunotherapy (ASIT) is also shown in this table.

### 3.2. Effect of ASIT Treatment on Percentages of Lymphocyte Subpopulations and on Level of Cytokines in AD Dogs

In the presented study, we aimed to evaluate how allergen-specific immunotherapy influenced the frequency of individual lymphocyte subpopulations in peripheral blood mononuclear cells (PBMC) and the level of cytokines involved in immune response to the allergens in plasma.

In the results described below, four groups are compared: control (healthy dogs), dogs with AD before treatment (0), dogs with AD after 3 months of ASIT (3 months) and dogs with AD after 6 months of ASIT (6 months).

Considering the varied percentage of cells of particular lymphocyte subpopulations and the concentration of cytokines in individual patients before ASIT and at subsequent stages of therapy, different immune responses to the administered allergens were observed in individual patients. Even so, the trend was similar.

**Figure 1:**
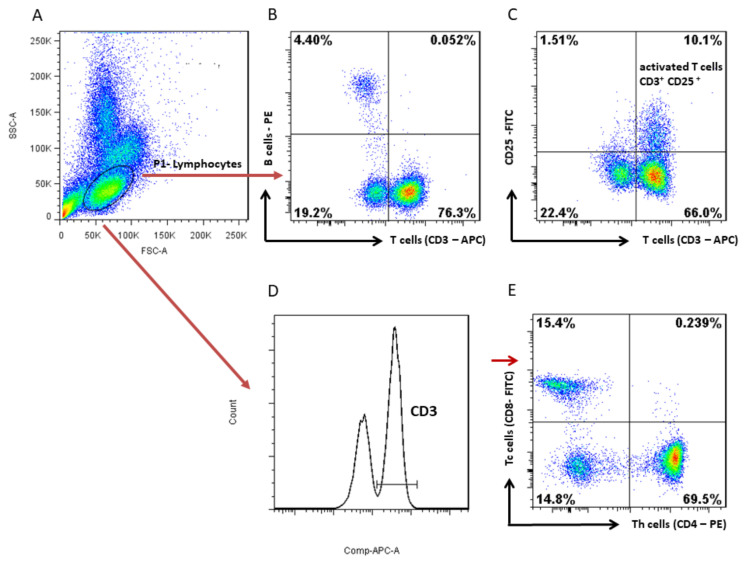
Representative plots of gating strategy for flow cytometry showing canine lymphocyte subpopulations. (**A**) Forward-scatter area (FS) versus side-scatter area (SS) plot to delineate the lymphocyte region. (**B**) B cells and T cells (CD3^+^) were gated among lymphocytes. (**C**) CD25 versus CD3 plot was used to gate activated T cells (CD3^+^CD25^+^) among lymphocytes. (**D**) Representative histogram of CD3^+^ cells, (**E**) among which Th cells (CD3^+^CD4^+^) and Tc cells (CD3^+^CD8^+^) were detected on CD4 versus CD8 plot. Data were analyzed using FlowJo software.

The results of T and B cell counts are expressed as the percentage of cells within the gating area of lymphocytes (P1), and the results of Th and Tc cell counts are presented as the percentage of CD3^+^ cells (Figure 1).

**Figure 2:**
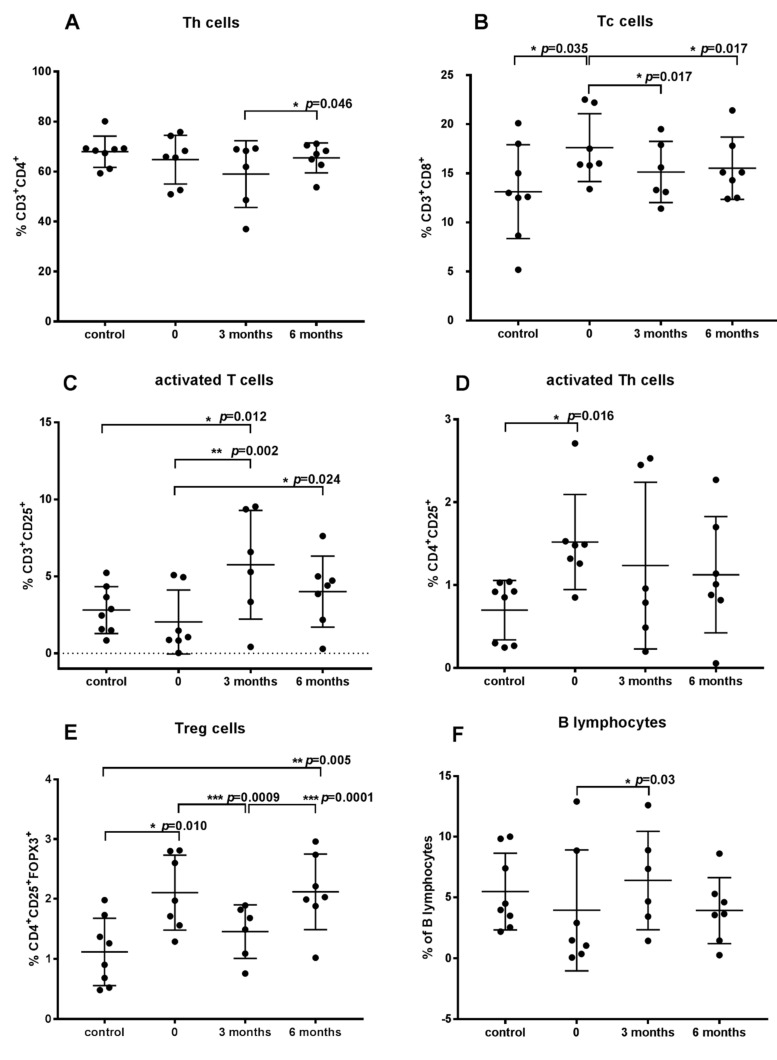
Percentage of lymphocyte subsets in peripheral blood of dogs with atopic dermatitis before ASIT (0), after 3 and 6 months of therapy, and in healthy dogs (control): (**A**) T helper cells, (**B**) T cytotoxic cells, (**C**) activated T lymphocytes, (**D**) activated Th cells, (**E**) Treg cells, (**F**) B lymphocytes. All data represent mean ± standard deviation. Symbols: *, **, *** represents the level of significance, respectively: *p*  <  0.05, *p* < 0.01, *p* < 0.001 (repeated measures ANOVA with Fisher’s post-hoc test).

There were no significant differences in the percentage of Th (CD3^+^CD4^+^) lymphocytes between the healthy group (68 ± 6.3) and the dogs with AD before therapy (64.8 ± 9.7) (Figure 2A). However, there was a significant difference in the percentage of Th cells in the dogs subjected to ASIT for 3 and 6 months: 59.0 ± 13.3 and 65.5 ± 5.9, respectively. After three months of therapy, the percentage of Th cells was the lowest. Dogs with AD prior to immunotherapy and during immunotherapy showed more Tc cells (CD3^+^CD8^+^) than the healthy dogs (Figure 2B). Dogs with AD before therapy demonstrated the highest percentage of Tc cells compared to the control group and compared to the values detected after 3 and 6 months of ASIT. Immunotherapy resulted in a significantly increased percent of B lymphocytes after 3 months of therapy, whereas after 6 months of ASIT the percent of B cells decreased to values lower than in the control group (Figure 2F). In addition, ASIT significantly influenced T cell activation, increasing the percentage of activated T cells (CD3^+^CD25^+^). After 6 months of treatment, the number of activated T cells decreased but was still higher than in the healthy group or AD group before ASIT (Figure 2C). In contrast, the percentage of activated Th cells (CD4^+^CD25^+^) was highest in dogs with AD before therapy compared to healthy animals and dogs during ASIT. On average, the percentage of activated Th cells was higher in dogs during treatment compared to healthy dogs (Figure 2D).

**Figure 3:**
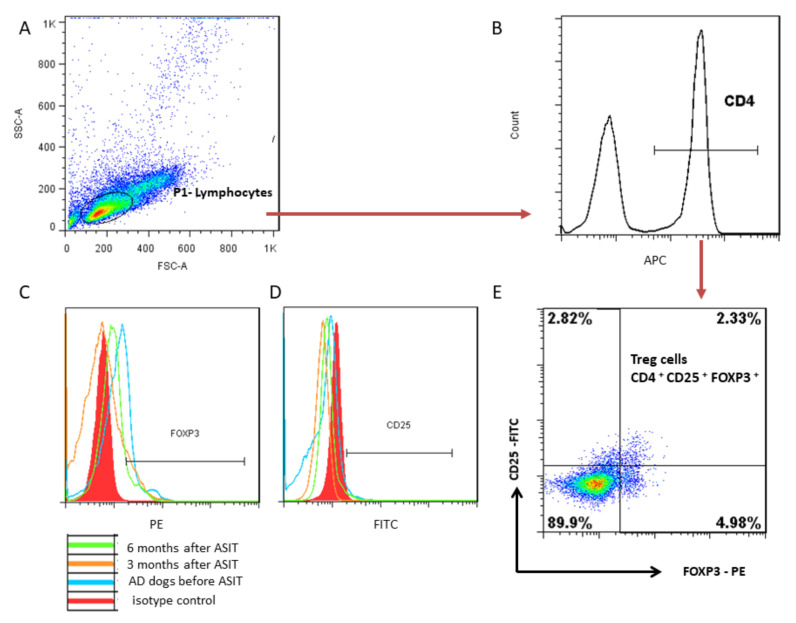
Representative plots of gating strategy for flow cytometry showing canine Treg characterization. (**A**) Forward-scatter area (FS) versus side-scatter area (SS) plot to delineate the lymphocyte region. (**B**) The CD4 population was gated among lymphocytes. (**C**,**D**) Representative histograms of CD25 and FOXP3 expression, respectively, for AD patients before ASIT and 3 and 6 months after ASIT, with isotypic control marked on the histograms. (**E**) CD25 versus FOXP3 plot was used to gate Treg cells (CD4^+^CD25^+^FOXP3^+^) among CD4^+^ T cells. Data were analyzed using FlowJo software.

The percentage of CD4^+^CD25^+^FOXP3^+^ (Treg) cells was quantified within the population of lymphocytes positively stained with anti-CD4^+^ antibody (Figure 3). The percentage of Treg (CD4^+^CD25^+^FOXP3^+^) cells in dogs with AD before treatment and after 6 months of therapy was the same, but was significantly higher than in the healthy group and in patients after three months of therapy. The percentage of Treg cells in dog after three months of ASIT was slightly higher than in healthy dogs (Figure 2E). Looking at the changes in the percentage of Treg cells in individual patients during ASIT, a decrease in Treg cells was observed in all patients after 3 months of therapy. However, after 6 months of therapy, the percentage of Tregs increased again in almost all patients, and in only one dog the amount of Tregs decreased even more. When comparing the values of the percentage of Tregs before treatment and 6 months after treatment in individual patients, a variable reaction of dogs to ASIT can be noted. In two dogs, the number of Tregs increased, in two dogs it decreased, and in three patients it was at a similar level (Figure S1). However, the mean values did not differ significantly before ASIT and after six months of the treatment (Figure 2E).

**Figure 4:**
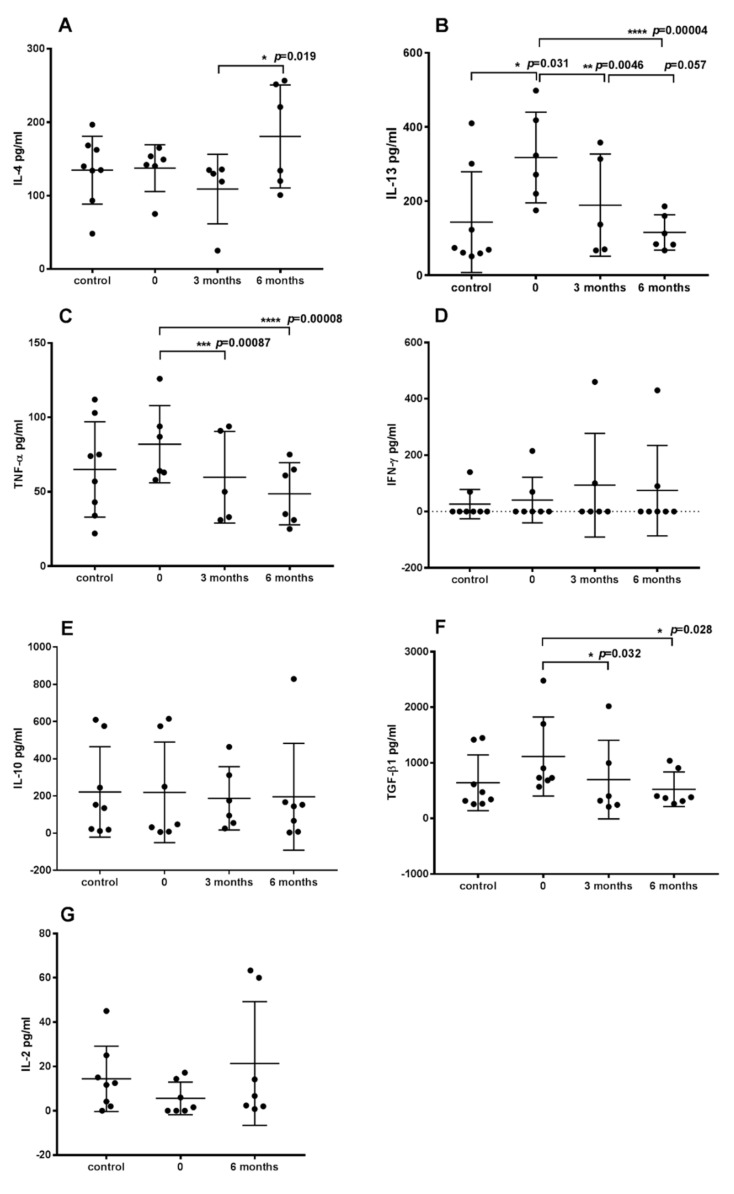
Concentration of cytokines (**A**) IL-13, (**B**) IL-4, (**C**) TNF-α, (**D**) IFN-γ, (**E**) IL-10, (**F**) TGFβ1 and (**G**) IL-2 in plasma of dogs with atopic dermatitis before ASIT (0), after 3 and 6 months of therapy, and in healthy dogs (control). All data represent the mean ± standard deviation. Symbols: *, **, ***, **** represent the level of significance, respectively: *p*  <  0.05, *p* < 0.01, *p* <0.001, *p* < 0.0001 (repeated measures ANOVA with Fisher’s post-hoc test).

The level of plasma cytokines, which play an important role in the immune response to allergens, was determined based on ELISA. The concentration of interleukins 4 and 13 secreted largely by Th2 cells was tested. The highest concentration of IL-13 was noted in cAD patients compared to the control group and to dogs during ASIT. The levels of IL-13 in plasma significantly decreased during treatment. After 6 months of therapy, the concentration of IL-13 in plasma was even lower than in healthy dogs (Figure 4A). An opposite effect was noted in the case of IL-4. The highest level of IL-4 in plasma was observed after 6 months of ASIT and differed significantly from the IL-4 level in the plasma of dogs after 3 months of treatment (Figure 4B).

The concentration of tested cytokines secreted by Th1 cells (IFN-γ and IL-2) did not differ significantly among groups. IFN-γ was only detected in two control dogs and two cAD patients before and during ASIT. In the remaining patients, IFN-γ was not detected (Figure 4D). IL-2 was not detected in one healthy dog and two AD dogs before treatment. There were no significant differences in IL-2 levels before and after 6 months of therapy, but a tendency of increased IL-2 levels was observed in all dogs after 6 months of therapy in comparison to cAD patients before ASIT (Figure 4G). In two patients, concentration of IL-2 increased only slightly (Figure S2).

The level of proinflammatory cytokine TNF-α was also tested. On average, higher levels of the cytokine were noted in atopic than in healthy dogs. The highest concentration of TNF-α was determined in AD dogs compared to treated dogs, and this result was significant. A reduction in TNF-α levels was observed after 6 months of treatment in all dogs (Figure 4C). The highest level of TNF-α in cAD patients before therapy may be associated with a greater number of Tc cells. Additionally, increased levels of IL-13 may indicate the strongest allergic reaction in AD dogs before treatment.

Despite the fact that an increased number of Treg cells was found, there was no difference in the level of IL-10 secreted by these cells. The concentration of IL-10 was similar in all studied groups (Figure 4E). On the other hand, the level of TGF-β, another cytokine secreted by Treg cells, which has been shown to have anti-inflammatory suppressive properties, differed significantly in dogs with AD before and after 3 and 6 months of therapy (Figure 4F).

## 4. Discussion

Allergen-specific immunotherapy (ASIT) is the only prophylactic and therapeutic procedure that is effective in many cases of human allergies. Moreover, it has been proven that in patients undergoing immunotherapy, in addition to alleviating symptoms or curing a specific allergy, the risk of acquiring another allergy is significantly reduced. It is now recognized that the key to the success of this form of treatment is the induction of allergen-specific lymphocytes with a regulatory (suppressive) function.

A more detailed understanding of the canine atopic dermatitis (cAD) mechanism, as well as the response to therapy, enriches veterinary knowledge, but it also gives a chance to learn more about the similarities and differences in response to allergens and immunotherapy in dogs and humans. These two species share the same environment, are inextricably linked, and show similarities in atopic dermatitis. In the presented experiment, we aimed to determine whether subcutaneous allergen-specific immunotherapy influenced the immune system response to allergens in dogs, and to identify the molecular mechanism of this response. After three months of therapy, no improvement was seen in any of the treated dogs. However, six out of seven dogs receiving ASIT showed a positive effect due to therapy after six months. Symptoms such as pruritus and skin lesions had decreased or disappeared. In five dogs, there was no need for pharmacological treatment during or after 6 months of therapy. One patient showed a hypersensitivity reaction and required prolongation of therapy due to administration of medications alleviating the allergic effect. Immunotherapy did not give a positive healing effect in this case.

Analyzing the results, it can be concluded that immunotherapy changed the number of individual lymphocyte subpopulations and concentration of secreted cytokines in peripheral blood of cAD patients subjected to immunotherapy. Nevertheless, in the results obtained, we did not observe a clear trend of changes, unlike what has been previously reported for human patients subjected to immunotherapy [24]. The differences in the obtained results may be due to the different duration of ASIT and a different method for determining the subpopulations of lymphocytes. Our study showed a significantly higher percentage of Treg cells (CD4^+^CD25^+^FOXP3^+^) in cAD patients before treatment compared to the healthy dogs. Similar results have also been obtained by other authors [15,17,18]. After three months of therapy, the percentage of Tregs significantly decreased, and after 6 months it increased significantly again (except for one patient). Nevertheless, changes in the number of Treg cells in individual patients differed. In two patients, the number of Tregs was significantly increased after 6 months of ASIT compared to before treatment. It is difficult to explain why the percentage of Tregs in all dogs decreased after three months of ASIT and then increased. Perhaps it is due to the fact that at the beginning of therapy, lower doses of allergens were administered, and from the 12th week of therapy, the dose increased to the highest, and therefore the percentage of suppressor Treg cells increased. Presumably a longer time (more than 6 months) of administration of the high dose of allergens would further increase the number of Treg cells. A study by Santos and coworkers [30] on exposure of beekeepers to hymenoptera venom showed that expression of FOXP3 in circulating Treg cells increases during exposure to a high dose of allergen (in this case bee venom) and was significantly higher during the beekeeping season compared to the inter-season. However, Keppel and coworkers [31] showed that the number of Treg cells in the peripheral blood of AD and healthy dogs was the same, and it increased from the third month of therapy. These data are not quite comparable due to the fact that Keppel and coworkers [31] defined the Treg cell population as CD4^+^FOXP3^+^, and we defined Treg by CD4^+^CD25^+^FOXP3^+^ expression. It is worth noting that in the case of B cells, there is exactly the opposite tendency, as after three months of therapy the highest number of B cells was observed.

In addition, the concentration of TGF-β in plasma was the highest in AD dogs before treatment. However, when looking at the results of individual patients, in two cases an increase in the level of this cytokine was observed after 6 months of therapy compared to the state before therapy (Figure S2G). A relationship can be noted between the highest percentage of Treg cells and the highest plasma concentration of TGF-β in AD dogs before therapy. This cytokine is secreted by Tregs but also stimulates the differentiation of Th0 cells towards Tregs. Although in the study by Martini and coworkers [32], no statistically significant difference in plasma levels of TGF-β was observed in AD dogs during subcutaneous immunotherapy (SCIT), a tendency of decreasing concentration of this cytokine during therapy could be noted, which is similar to our results.

In the presented study, the average plasma level of IL-10 was similar in cAD patients during ASIT and before therapy and did not differ from that of the healthy dogs. Further, Martini and coworkers [32] did not show changes in the plasma level of IL-10 during SCIT after 3, 6 and 12 months of application. However, when looking at the results of individual patients in our study, it can be noted that in three dogs, the level of IL-10 increased after 6 months of treatment compared to before therapy (Figure S2F). Keppel et al. [31] observed that the level of IL-10 increased after 6 months of therapy, while before and after 3 months of treatment it was the same as in healthy dogs. It is possible that in dogs included in the present study, the concentration of IL-10 in plasma could have also increased after prolonged use of ASIT. Human studies point to the role of inducible Treg (iTreg) cell CD4^+^FOXP3^−^ IL-10-producing Tr1 cells in maintaining a healthy immune response [33,34]. In the case of dogs, the presence of this type of regulatory cells has not been tested so far. Perhaps after prolonged immunotherapy in dogs, the increase in IL-10 is associated with the formation of Tr1 cells mediating antigen-specific T cell tolerance.

Immunotherapy has been associated with a shift from T helper cell type-2 (Th2) immune responses, which are associated with the development of atopic conditions, to a better balance, with more Th1 immune responses [24]. This tendency can be observed partially in our research. The decrease in IL-13 levels in plasma during therapy suggests a decrease in Th2 activity. It is surprising, however, that in four patients, the level of IL-4 (interleukin also secreted by Th2 cells) increased after 6 months of ASIT. Interestingly, the level of IL-13 decreased in these patients (Figure S2A,B). The level of IL-4 was comparable in the control dogs and cAD patients before ASIT. Th1 cell activity is even more difficult to determine. IFN-γ was detected in only two AD dogs and in two control dogs. In one patient, the level of this cytokine increased during therapy, and in the other dogs it decreased (Figure S2D). Shida and coworkers [35] stated that levels of IFN-γ and IL-4 mRNA were lower in atopic dogs compared with non-atopic controls, and, after immunotherapy, the level of IFN-γ was significantly higher than before, whereas the level of IL-4 mRNA was not changed.

In the case of IL-2, an increase in the level of this cytokine was observed after 6 months of ASIT in all dogs compared to the level before therapy, and at the same time an increase in the number of activated T cells (CD3^+^CD25^+^) was noted, which indicates increased expression of the α-chain of the interleukin 2 receptor (CD25). It is worth noting that in three cAD patients, IL-2 was undetectable before therapy. These results seem to confirm the beneficial effect of immunotherapy. IL-2 is secreted by activated CD4^+^ T cells, although Th1 cells are believed to be the major source of this cytokine [36]. It should be emphasized that IL-2 signaling is requisite for the development and survival of Treg cells, directly regulating FOXP3 in human and murine Tregs and enhancing the expansion of these cells in vivo [37,38,39].

Additionally, during immunotherapy, the level of proinflammatory cytokine TNF-α was decreased compared to the concentration detected in cAD patients before therapy. TNF-α is secreted at the early stage of allergen sensitization and then continues to promote the inflammation cascade in the effector phase of allergic reactions. It has the capacity to promote Th2 responses [40,41]. Further, the significantly highest percentage of Tc cells (CD8^+^) was observed in cAD patients before ASIT compared to healthy dogs and patients during treatment. Verde and coworkers [42] also found a higher percentage of CD8^+^ cells in atopic dogs compared to healthy dogs. Hemino and coworkers [12,13] stated that CD8^+^ cells were essential for the development of AD skin inflammation in both mice and humans, and these cells were involved in the initiation of skin inflammation. The reduction of the TNF-α level and the reduction of the number of Tc cells (CD8^+^) seem to confirm the beneficial effect of the applied ASIT therapy.

We still know too little about cAD compared to human AD to conclude that dogs can be a model for humans in AD research. Nevertheless, more detailed understanding of the immune response of dogs to allergens as well as to ASIT is beneficial for comparative medicine. Results of the present study confirm the beneficial effect of the applied allergen-specific immunotherapy in cAD. Six out of seven dogs receiving ASIT showed a positive effect of the therapy. Immunotherapy influenced the number of cells in specific lymphocyte subpopulations and affected the levels of cytokines detected in the peripheral blood of cAD patients subjected to immunotherapy. Summarizing the results, it should be noted that reduced levels of IL-13 and TNF-α along with the changes observed in the number of the T cells—reduction of Tc cells (CD8^+^) and an increased number of activated T cells (CD3^+^CD25^+^)—confirm the beneficial effect of the applied ASIT therapy. ASIT and its duration induced changes in the number of Treg cells in cAD patients. However, it remains unclear why the number of Treg cells after three months of therapy decreased compared to before therapy and then after 6 months increased to the level before therapy or even higher. It should be emphasized that despite the significant changes in the lymphocyte subpopulations and in the level of cytokines observed among the tested AD dogs after ASIT, the response of individual patients to therapy can vary significantly.

## Figures and Tables

**Table 1:** Description of patients taking part in experiment: breed, age, clinical symptoms before allergen-specific immunotherapy (ASIT) and reaction to therapy after 13 and 29 weeks.

Breeds	Age Years/ Months	Clinical Symptoms	1st Stage of Immunotherapy after 13 Weeks	2nd Stage of Immunotherapy after 29 Weeks	Commentary
Labrador retriever	4/5	Dermatitis on the bridge of the nose; severe seasonal pruritus; no other skin lesions	No improvement—the dog was still itchy and rubbing his face	Significant improvement was observed at the end of this phase; no additional treatment was needed	No antipruritic drugs were used during Phases I and II of immunotherapy
American Staffordshire terrier	2/2	Recurrent papular dermatitis in the lumbosacral area; erythema and papules in the groin region and on the lateral surface of hind limbs; severe pruritus; good response to steroid treatment	Hypersensitivity reactionafter each dose of the allergen extract, which showed follicular dermatitis, erythema, severe pruritus	Similar reactions as in Phase I	The duration of whole immunotherapy was prolonged due to hypersensitivity reactions that occurred after each allergen dose and required treatment. The interval between allergen doses was extended by the time of each treatment
American Staffordshire terrier	6/5	Erythema in the groin area and on the medial surface of front limbs	Continuation of immunotherapy; clinical examination of the patient was not possible during this time; lack of feedback from the owner	There was significant improvement after discontinuation of immunotherapy	No antipruritic drugs were used during the whole immunotherapy
Labrador retriever	2	Severe pruritus; seborrheic interdigital dermatitis (front limbs)	Persisting pruritus and seborrheic dermatitis	Initial improvement was observed—the dog stopped licking his paws excessively and was less pruritic. Good response to immunotherapy—the dog was no longer pruritic, even after discontinuation of immunotherapy	No antipruritic drugs were used during the whole immunotherapy
Small Münsterländer	3	Recurrent pododermatitis of the front limbs (interdigital spaces); recurrent ceruminous otitis; erythema of the concave pinna surface in both ears; no pustules or papules on the skin	No changes in the clinical picture were observed (no improvement or exacerbation of clinical signs)	There was significant reduction in pruritus; no skin changes were noted after the last allergen dose. Good response to immunotherapy	No antipruritic drugs were used during the whole immunotherapy
Golden retriever	5	Erythema and crusts on the lower abdomen; chronic ceruminous otitis; epidermal collarettes; severe pruritus	Epidermal collarettes in the groin area as well as ceruminous otitis were still observed	Persisting epidermal collarettes; recurrent otitis externa; seborrheic dermatitis. However, there was significant reduction in pruritus after discontinuation of immunotherapy	There was a need to use steroids (dexamethasone injections) and/or antibiotics (cefalexin) to treat pruritic skin lesions during both stages of immunotherapy
Golden retriever	3/6	Erythema of interdigital spaces and dorsal surface of front limbs; lower abdomen erythema; pustules on the skin of right thigh; otitis externa (only right ear)	Initial exacerbation of pruritus and dermatitis. Spontaneous resolution of clinical signs was observed later without any treatment needed	Occasional erythema of the inner surface of front limbs was observed. Significant improvement in dog’s general skin condition	The dog was less pruritic and did not need any additional antipruritic treatment

## Data Availability

The data are contained within the article and Supplementary Materials.

## Supplementary Materials

The following supporting information is available online at: https://www.mdpi.com/article/10.3390/vaccines10071037/s1, Figure S1. Effect of ASIT on percentage of Treg cells in peripheral blood of individual atopic dermatitis dogs before therapy (0) and after 3 and 6 months of therapy. Figure S2. Effect of ASIT on changes in concentration of cytokines (IL-4, IL-13, TNF-α, IFN-γ, IL-10, TGF-β1, IL-2) in plasma of individual cases during ASIT: atopic dermatitis before therapy (0) and after 3 and 6 months of therapy
