# Supplementary figures and images for: Cytokine and Lymphocyte Profiles in Dogs with Atopic Dermatitis after Allergen-Specific Immunotherapy
Source: Vaccines (Basel). 2022 Jun 28;10(7):1037. doi: 10.3390/vaccines10071037 (PMC9323343; doi:10.3390/vaccines10071037)

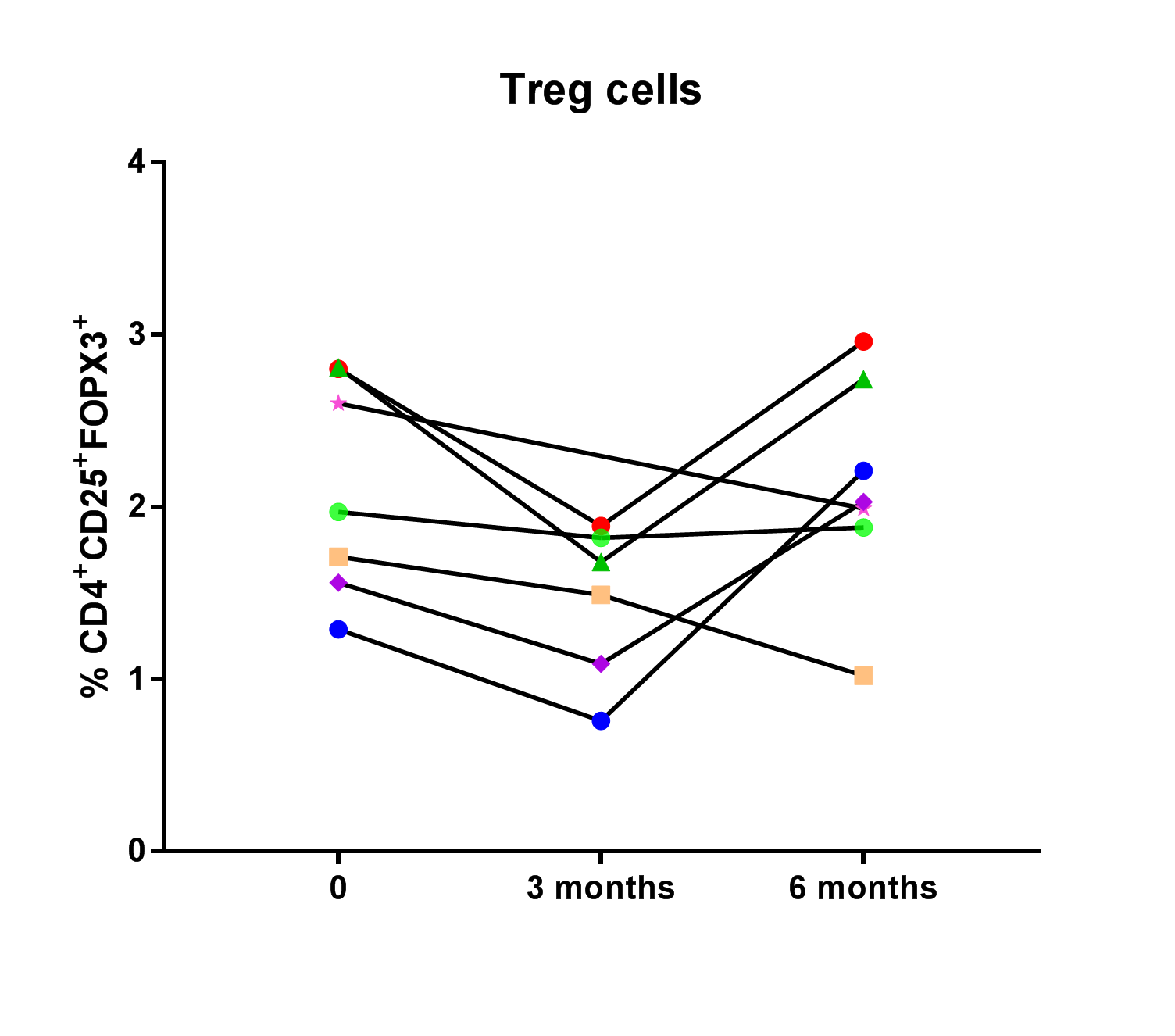

Supplement: Supplementary file 1 [file vaccines-10-01037-s001.zip › Figure S1.tif]

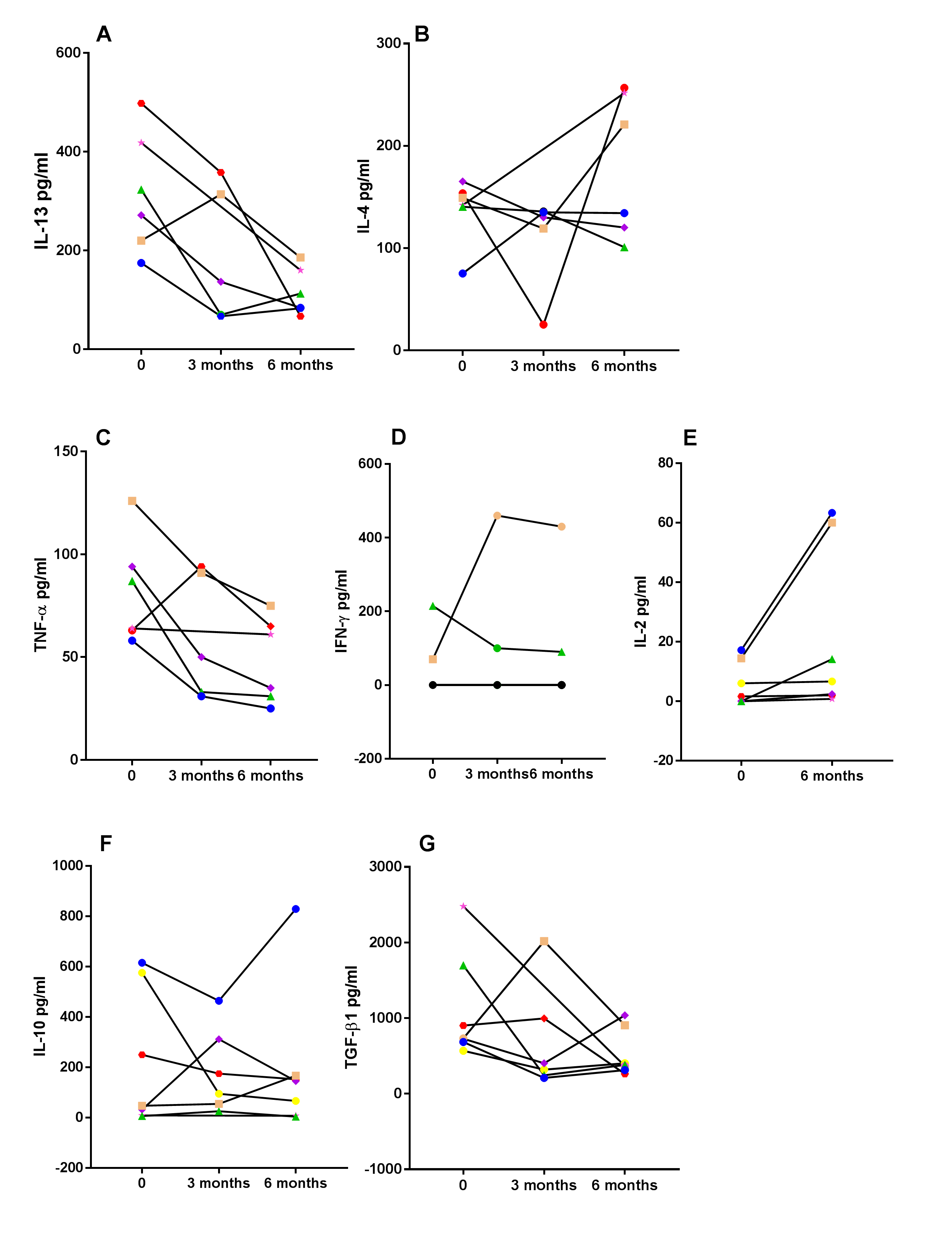

Supplement: Supplementary file 1 [file vaccines-10-01037-s001.zip › Figure S2.tif]
